# Analysis of cellulose synthesis in a high-producing acetic acid bacterium *Komagataeibacter hansenii*

**DOI:** 10.1007/s00253-023-12461-z

**Published:** 2023-03-17

**Authors:** Martin Bimmer, Martin Reimer, Andreas Klingl, Christina Ludwig, Cordt Zollfrank, Wolfgang Liebl, Armin Ehrenreich

**Affiliations:** 1 School of Life Sciences, Technical University of Munich, Emil-Ramann-Straße 4, 85354 Freising, Germany; 2 Technical University of Munich, Campus Straubing, Schulgasse 16, 94315 Straubing, Germany; 3 Plant Development, Ludwig-Maximilans-Universität München, Großhaderner Str.2, 82152 BiozentrumPlanegg-Martinsried, Germany; 4 Bavarian Center for Biomolecular Mass Spectrometry (BayBioMS), School of Life Sciences, Technical University of Munich, Gregor-Mendel-Straße 4, 85354 Freising, Germany

**Keywords:** *Komagataeibacter hansenii*, Bacterial cellulose, Markerless deletion, Acetic acid bacteria, Cellulose fiber, Proteomic analysis

## Abstract

**Abstract:**

Bacterial cellulose (BC) represents a renewable biomaterial with unique properties promising for biotechnology and biomedicine. *Komagataeibacter hansenii* ATCC 53,582 is a well-characterized high-yield producer of BC used in the industry. Its genome encodes three distinct cellulose synthases (CS), *bcsAB1*, *bcsAB2*, and *bcsAB3*, which together with genes for accessory proteins are organized in operons of different complexity. The genetic foundation of its high cellulose-producing phenotype was investigated by constructing chromosomal in-frame deletions of the CSs and of two predicted regulatory diguanylate cyclases (DGC), *dgcA* and *dgcB*. Proteomic characterization suggested that BcsAB1 was the decisive CS because of its high expression and its exclusive contribution to the formation of microcrystalline cellulose. BcsAB2 showed a lower expression level but contributes significantly to the tensile strength of BC and alters fiber diameter significantly as judged by scanning electron microscopy. Nevertheless, no distinct extracellular polymeric substance (EPS) from this operon was identified after static cultivation. Although transcription of *bcsAB3* was observed, expression of the protein was below the detection limit of proteome analysis. Alike BcsAB2, deletion of BcsAB3 resulted in a visible reduction of the cellulose fiber diameter. The high abundance of BcsD and the accessory proteins CmcAx, CcpAx, and BglxA emphasizes their importance for the proper formation of the cellulosic network. Characterization of deletion mutants lacking the DGC genes *dgcA* and *dgcB* suggests a new regulatory mechanism of cellulose synthesis and cell motility in *K. hansenii* ATCC 53,582. Our findings form the basis for rational tailoring of the characteristics of BC.

**Key points:**

• *BcsAB1 induces formation of microcrystalline cellulose fibers.*

• *Modifications by BcsAB2 and BcsAB3 alter diameter of cellulose fibers.*

• *Complex regulatory network of DGCs on cellulose pellicle formation and motility.*

**Supplementary Information:**

The online version contains supplementary material available at 10.1007/s00253-023-12461-z.

## Introduction

Bacterial cellulose (BC) is a biodegradable and sustainable biomaterial with multiple applications (Cazón and Vázquez 2021; Klemm et al. 2021) and has been generating growing revenues on the market over the past several years (Reports&Data 2021). The high purity of BC and its microcrystalline characteristics makes BC an interesting alternative to cellulose of plant origin (Choi and Shin 2020). In contrast to the cellulose synthesized by plants that accounts by far for the largest amount of cellulose processed by industry, BC is free of non-cellulosic polymers like lignin or hemicellulose (Cannon and Anderson 1991; Gullo et al. 2018), while plant cellulose must be purified under costly and harsh chemical conditions (Saedi et al. 2021). Especially acetic acid bacteria are well-known as high-level producers of microcrystalline cellulose, and this aspect was thoroughly studied (Abidi et al. 2021; Buldum and Mantalaris 2021; Gullo et al. 2018). The unique properties of high-water absorption and high tensile strength define the different applications of BC. Also, many approaches are done to adopt the structure and unique features of BC on molecular level (Manan et al. 2022). In biomedicine, BC can be used in many areas, such as tissue engineering, wound dressing, or pharmaceutical applications (Klemm et al. 2021; Portela et al. 2019; Ullah et al. 2016). BC is generally recognized as safe (GRAS) by the American Food and Drug Administration (FDA) since 1992 and is used as a thickening agent in the food industry. It represents an important constituent of the traditional Philippine dessert *nata de coco*. Among other strains, a “*Komagataeibacter xylinum*” strain is used by CP Kelco U.S. Inc. (San Diego, USA) for the industrial scale production of a commercial BC suspension.

For acetic acid bacteria, the cellulose biofilm, called “mother of vinegar,” creates a niche of high substrate and oxygen supply in their natural habitats. *K.* *hansenii* ATCC 53,582 (formerly known as *K. xylinus* or *Gluconacetobacter xylinus* and recently suggested to be reclassified as *Novacetimonas hansenii* (Brandão et al. 2022)) is characterized by an exceptionally high cellulose production rate among cellulose-producing acetic acid bacteria strains (Fang and Catchmark 2015). This capacity makes the strain one of the most important strains for BC production in biotechnology. This strain is also the object of a patent (Brown Jr 1990). Although many aspects of BC synthesis have been studied before, only a few studies have focused on the genetics of cellulose formation (Ryngajłło et al. 2020). Until recently, efficient techniques for the generation of markerless modifications in the genome of cellulose-producing strains were not available. Most genetic studies were conducted by transposon mutagenesis, which limits the interpretability of the results due to polar effects and other artefacts (Buldum and Mantalaris 2021). Besides our previous study on general genetic aspects of cellulose synthesis in *K.* *hansenii* ATCC 23,769 (Bimmer et al. 2022), the present study focused on the highly productive and biotechnologically relevant strain *K.* *hansenii* ATCC 53,582. We sought to identify possible reasons for its high cellulose yield by comparing different strains and analyzing differences of their cellulose synthase (CS) operons. The genome sequence of *K.* *hansenii* ATCC 53,582 contains three (putative) CS operons (*bcsABCD*) with different numbers and arrangement of genes (Florea et al. 2016; Römling and Galperin 2015; Wong et al. 1990). But the accessory proteins CmcAx (carboxymethyl cellulase), CcpAx (cellulose complementing factor), and BglxA (putative cellulose deacylase) are only present once, close to the major operon encoding BcsAB1. The BC synthase complex comprises the fused protein BcsAB in the inner membrane, BcsC forming an extrusion pore in the outer membrane and BcsD for correction of errors of the growing glucan chain in the periplasm (Du et al. 2016; Sunagawa et al. 2013). CmcAx is an extracellular cellulase (Standal et al. 1994), while the other accessory proteins (CcpAx and BglxA) are localized in the periplasm (Sunagawa et al. 2013). According to the complexity of the operons, we named the CSs according to their assumed importance: *bcsAB1* as “major CS”; *bcsAB2* as “minor CS” with; and *bcsAB3* as “accessory CS.” These designations are also used below.

Transcription analysis of the CS operons by RT-qPCR, as well as label-free quantitative proteomics targeting the CSs and the associated proteins CmcAx, CcpAx, and BglxA, are useful experimental techniques that can be employed to draw an improved picture of the expression of transcription and translation in the context of cellulose synthesis. We sought to resolve the function of each of the three CS genes (*bcsAB1* (ATCC53582_00602), *bcsAB2* (ATCC53582_02530), and *bcsAB3* (ATCC53582_01242)) and of their respective operons. Therefore, chromosomal in-frame deletions of each CS were constructed, first one by one, then simultaneously, to enable the characterization of the effects of the major CS BcsAB1, as well as the minor and the accessory CS on the cellulose network and on the overall cellulose yield. Furthermore, the genome of *K.* *hansenii* ATCC 53,582 encodes an acetyl transferase (*bcsY*, ATCC53582_02528) as part of the minor CS operon. Deletion of *bcsY* should be a useful strategy to identify the contribution of the acetyl transferase BcsY, as acetylated cellulose has not been described in previous studies. Cyclic-di-GMP formed by diguanylate cyclases (DGCs) is the second messenger inducing cellulose formation by binding the PilZ-domain of *bcsA*. Thus, it is also of special interest to use clean genome deletion methods to gain a better understanding of the roles of two predicted regulatory DGCs *dgcA* (ATCC53582_00180) and *dgcB* (ATCC53582_02359) for high-level cellulose production (Amikam and Galperin 2005; Ross et al. 1990).

In the present study, we report the construction of various mutants, their phenotypical, transcriptomic, and proteomic characterization as well as the quantification and analysis of the synthesized cellulose by scanning electron microscopy and physicochemical parameters.

## Materials and Methods

### Bacterial strains, plasmids, and growth conditions

*K. hansenii* ATCC 53,582 was purchased from the American Type Culture Collection (Manassas, VA, USA). All bacterial strains are listed in Table 1. *E. coli* Top10 strains were grown in lysogeny broth (Lennox) medium (LB) at 37 °C and 180 rpm containing 50 µg/mL kanamycin. *K.* *hansenii* ATCC 53,582 was grown at 30 °C, 180 rpm in complex medium (1.5% (w/v) yeast extract, 0.05% (w/v) glycerol and 8% d-mannitol, pH 6.0) for growth and selection of integration. For counter-selection, the content of yeast extract was reduced to 0.015% (w/v) and 0.5% (w/v) of casamino acids were added as well as 120 µg/mL 5′-fluoro-cytosine (TCI, Zwijndrecht, Belgium) (Bimmer et al. 2022). For proteomic and transcriptional analysis, the strains were grown from single colonies in presence of 6 U/mL cellulase from *Trichoderma reesei* (Sigma Aldrich, Steinheim, Germany) in baffled flasks and 10 mL HS medium at 30 °C and 60 rpm for 3 days. The used oligonucleotides were synthesized by Eurofins Genomics GmbH (Ebersberg, Germany) and are listed in Table 2.

**Table 1:** Strains used and generated in this study

Strain	Properties	Reference/source
*Escherichia coli*		
*E. coli* TOP10	F^*−*^, *mcrA* Δ(*mrr-hsd*RMS-*mcr*BC), φ80*lac*ZΔM15, Δ*lac*X74, *nupG*, *recA1*, *ara*D139 Δ(ara-leu)7697, *galE*15, *galK*16, *rpsL*(Str^R^), *end*A1 λ^−^	Invitrogen (CA, USA)
*E. coli* HB101	F^−^, *hdsS*20 (r-B, m-B), *supE*44, *ara*-14, *galK*-2, lacY1, *proA*2, *rpsL*20, *xyl*-5, *mtl*-1, *recA*13, *Km*^*R*^, *oriColE*1, *RK*2-Tra + , *mH*-1 carrying plasmid pRK2013	Boyer and Roulland-Dussoix (1969); Figurski and Helinski (1979)
*Komagataeibacter hansenii*		
*K. hansenii* ATCC 53,582	Cellulose^+^, FC^R^, Cef^R^	American Type Culture Collection
*K. hansenii* Δ*bcsAB1*	Deletion of *bcsAB1* (ATCC53582_00602)	This work
*K. hansenii* Δ*bcsAB2*	Deletion of *bcsAB2* (ATCC53582_02530)	This work
*K. hansenii* Δ*bcsAB3*	Deletion of *bcsAB3* (ATCC53582_01242)	This work
*K. hansenii* Δ*bcsAB1* Δ*bcsAB2*	Single expression *of bcsAB3*	This work
*K. hansenii* Δ*bcsAB1* Δ*bcsAB3*	Single expression *of bcsAB2*	This work
*K. hansenii* Δ*bcsAB2* Δ*bcsAB3*	Single expression *of bcsAB1*	This work
*K. hansenii* Δ*bcsAB1* Δ*bcsAB2* Δ*bcsAB3*	Deletion of *bcsAB1*, *bcsAB2* and *bcsAB3*	This work
*K. hansenii* Δ*bcsY*	Deletion of *bcsY* (ATCC53582_02528)	This work
*K. hansenii* Δ*dgcA*	Deletion of *dgcA* (ATCC53582_00180)	This work
*K. hansenii* Δ*dgcB*	Deletion of *dgcB* (ATCC53582_02359)	This work

**Table 2:** Primers used in this study

Name	Sequence (5′–3′)	Application
acsAB_F	CGTTGACGCTGCTCGTTTACATC	Check primer for *bcsAB1*
acsAB_R	CGGGTGTAACCGGTAACCATGAC	
acsAB2_F	ATCACGGCCACCATGCATAAC	Check primer for *bcsAB2*
acsAB2_R	GTGTCGCTGAGATAGCCGAAC	
acsAB3_F	AGCGATGGTCCCAATACTTC	Check primer for *bcsAB1*
acsAB3_R	CCTTCGGCTTCATGGATCTTAC	
acsAB_seq_F	CGCACATCAGCATCGTCCATAAG	Sequencing primer for *bcsAB1*
acsAB_seq_R	CAGCCAGCGTCTGGTAATATTC	
acsAB2_seq_F	CAGTACCGACAGGAGCGAATC	Sequencing primer for *bcsAB2*
acsAB2_seq_R	GGGCCTGTTAACCCTTAAAGC	
acsAB3_seq_F	CTTCGCCTCCAATCTAAGGTG	Sequencing primer for *bcsAB3*
acsAB3_seq_R	CCCTCCTCTATCTGCTCATTG	
Fla A-SL_AB1_F	CTTCCGGCTCGTATGTTGTGTTCGATCAGGCGGAGCATGACAC	Upstream flank of *bcsAB1* for SLiCE
Fla A-SL_AB1_R	GAAGCATATCGTTTATGGGTCACAGGGACAGGCGGTCGCCTGAATA	
Fla B-SL_AB1_F	TATTCAGGCGACCGCCTGTCCCTGTGACCCATAAACGATATGCTTC	Downstream flank of *bcsAB1* for SLiCE
Fla B-SL_AB1_R	CCAGTCTAGCTATCGCCATGTCCTTGGCATTCAGGTTCAGTG	
pKOS6b_SL-AB1_F	CACTGAACCTGAATGCCAAGGACATGGCGATAGCTAGACTGG	Backbone pKOS6b for SLiCE *bcsAB1*
pKOS6b_SL-AB1_R	GTGTCATGCTCCGCCTGATCGAACACAACATACGAGCCGGAAG	
Fla A-SL_AB2_F	CTTCCGGCTCGTATGTTGTGGTTGCATCGGCCTGTATTTCAC	Upstream flank of *bcsAB2* for SLiCE
Fla A-SL_AB2_R	GCTGTCCCCGGGGATTTGAAGGTACGATGCGTCGGGGGATATGTCCG	
Fla B-SL_AB2_F	CGGACATATCCCCCGACGCATCGTACCTTCAAATCCCCGGGGACAGC	Downstream flank of *bcsAB2* for SLiCE
Fla B-SL_AB2_R	CCAGTCTAGCTATGCCATGTGCATGTTGAGCGAGGCATAGTG	
pKOS6b_SL-AB2_F	CACTATGCCTCGCTCAACATGCACATGGCGATAGCTAGACTGG	Backbone pKOS6b for SLiCE *bcsAB2*
pKOS6b_SL-AB2_R	GTGAAATACAGGCCGATGCAACCACAACATACGAGCCGGAAG	
Fla A-SL_AB3_F	CTTCCGGCTCGTATGTTGTGGCACCATACTCCATAGCGTAAC	Upstream flank of *bcsAB3* for SLiCE
Fla A-SL_AB3_R	CATACATGGTCGGTTTCCTTCTGTTAATATGCCGGTTTAAAGGATAATC	
Fla B-SL_AB3_F	CCAGTCTAGCTATCGCCATGTCGACGCATTTCGCCATCATCTG	Downstream flank of *bcsAB3* for SLiCE
Fla B-SL_AB3_R	GATTATCCTTTAAACCGGCATATTAACAGAAGGAAACCGACCATGTATG	
pKOS6b_SL-AB3_F	CAGATGATGGCGAAATGCGTCGACATGGCGATAGCTAGACTGG	Backbone pKOS6b for SLiCE *bcsAB3*
pKOS6b_SL-AB3_R	GTTACGCTATGGAGTATGGTGCCACAACATACGAGCCGGAAG	
DgcA_F	CTAACAGGGCTGTCAGATAGG	Check primer for *dgcA*
DgcA_R	GATCCTGTTCCTGTGGATACTG	
DgcA_seq_F	CCGCTCCTTGGTGTTCATCAG	Sequencing primer for *dgcA*
DgcA_seq_R	ACGGCCCTGTACCCAATATCG	
Fla A-SL_DgcA_F	CTTCCGGCTCGTATGTTGTGTGCACATCTTCCAGCGTATC	Upstream flank of *dgcA* for SLiCE
Fla A-SL_DgcA_R	CTTTCTCCCCCTGAGGTTGATGAAGTCTAAGGCAACATTCCAG	
Fla B-SL_DgcA_F	CTGGAATGTTGCCTTAGACTTCATCAACCTCAGGGGGAGAAAG	Downstream flank of *dgcA* for SLiCE
Fla B-SL_DgcA_R	CCAGTCTAGCTATCGCCATGTTGTTTGCCCTTGCCTATGTGAC	
pKOS6b_SL-DgcA_F	GTCACATAGGCAAGGGCAAACAACATGGCGATAGCTAGACTGG	Backbone pKOS6b for SLiCE *dgcA*
pKOS6b_SL-DgcA_R	GATACGCTGGAAGATGTGCACACAACATACGAGCCGGAAG	
DgcB_seq_F	GTCGGAATGCACGATATACC	Sequencing primer for *dgcB*
DgcB_seq_R	TTGCTTGAGGAGCTGAAGTG	
DgcB_F	CGGACATCCTGCATAGAATTGG	Check primer for *dgcB*
DgcB_R	CCGTCCTACAGCAACCATATC	
Fla A-SL_DgcB_F	CTTCCGGCTCGTATGTTGTGCGGCCATGACATGTGGTTTCC	Upstream flank of *dgcB* for SLiCE
Fla A-SL_DgcB_R	CGTTTTCAGGTCTGAGGGAAACGCACCATGACGCAGGGTGAT	
Fla B-SL_DgcB_F	ATCACCCTGCGTCATGGTGCGTTTCCCTCAGACCTGAAAACG	Downstream flank of *dgcB* for SLiCE
Fla B-SL_DgcB_R	CCAGTCTAGCTATCGCCATGTGGGATGCTCAACCTGCATTAC	
pKOS6b_SL-DgcB_F	GTAATGCAGGTTGAGCATCCCACATGGCGATAGCTAGACTGG	Backbone pKOS6b for SLiCE *dgcB*
pKOS6b_SL-DgcB_R	GGAAACCACATGTCATGGCCGCACAACATACGAGCCGGAAG	
bcsY_F	CCGACACGATATAGGGATGG	Check primer for *bcsY*
bcsY_R	ACAGGCTGACAAGCTATCG	
Fla A-SL_bcsY_F	CTTCCGGCTCGTATGTTGTGCGGTATCCACATCCGCATTCACG	Upstream flank of *bcsY* for SLiCE
Fla A-SL_bcsY_R	GTGAAATACAGGCCGATGCAACAGCGTGCGGGGCGATGTACC	
Fla B-SL_bcsY_F	GGTACATCGCCCCGCACGCTGTTGCATCGGCCTGTATTTCAC	Downstream flank of *bcsY* for SLiCE
Fla B-SL_bcsY_R	CCAGTCTAGCTATCGCCATGTGGATGTGGCTGGACTGGTATCTC	
pKOS6b_SL-bcsY_F	GAGATACCAGTCCAGCCACATCCACATGGCGATAGCTAGACTGG	Backbone pKOS6b for SLiCE *bcsY*
pKOS6b_SL-bcsY_R	CGTGAATGCGGATGTGGATACCGCACAACATACGAGCCGGAAG	

### Cellulose formation

Cultures for cellulose formation were grown without agitation in HS medium (5% (w/v) yeast extract, 0.5% (w/v) peptone, 0.12% (w/v) citric acid 1-hydrate, 0.35% (w/v) disodium phosphate 2-hydrate, and 2% d-glucose, pH = 5.0) at 25 °C (Hestrin and Schramm 1954). Single colonies of the high-yield cellulose-producing strain *K.* *hansenii* ATCC 53,582 were inoculated in jars with 250 mL HS medium and incubated under static conditions without agitation at 25 °C for 6 days. The cellulose formed at the surface was used for Fourier transform infrared spectroscopy (FTIR) and X-ray crystallography (XRD) as well as the measurement of the mechanical strength, dry mass, and scanning electron microscopy (SEM). An extended cultivation time of 14 days helped to better illustrate the phenotypical differences observed by eye (see corresponding images in the “Results” section).

### DNA techniques and genetic manipulation

DNA was isolated using the MasterPure DNA purification kit (Epicenter, Madison, WI, USA) and resolved in ddH_2_O. The NucleoSpin® Plasmid EasyPure kit (Macherey–Nagel, Düren, Germany) was used according to the manufacturer’s protocol. PCR fragments used in the course of genomic modification of strains were amplified using the S7 Fusion High-Fidelity DNA Polymerase (Biozym Scientific GmbH, Hessisch Oldendorf, Germany). Larger fragments were amplified using PrimeSTAR® Max DNA Polymerase (TAKARA BIO INC., Kusatsu, Japan). For subsequent cloning and sequencing of PCR fragments, the products were purified with NucleoSpin® PCR clean-up gel extraction kit (Macherey–Nagel, Düren, Germany). Constructs were checked by analytical PCR using Phire II Hot Start DNA Polymerase (Thermo Fisher Scientific, Waltham, MA, USA). Direct analysis of clones was done by colony PCR (Güssow and Clackson 1989). Plasmids for chromosomal clean deletions were constructed using the SLiCE ligation technique (Zhang et al. 2014). The previously well-established plasmid pKOS6b (Kostner et al. 2013) was used as vector backbone. For construction of the deletion plasmid pKOS6b_ΔbcsAB1_53582, the upstream flanking region was amplified using the primer pair Fla A-SL_AB1_F and Fla A-SL_AB1_R, while Fla B-SL_AB1_F and Fla B-SL_AB1_R were used to amplify the downstream flanking region. pKOS6b_SL-AB1_F and pKOS6b_SL-AB1_R were used to amplify the plasmid backbone. The other deletion plasmids were constructed analogously using the primers as listed in Table 2. Fast-digest restriction enzymes purchased from Thermo Fisher Scientific (Waltham, MA, USA) were used for restriction digestion.

### Transformation and genetic modification of *K. hansenii* ATCC 53,582

The clean deletion system for acetic acid bacteria used here is based on *codBA* counter-selection as described by Kostner et al. (2013). The same technique was also successfully used in *Bacillus licheniformis* (Kostner et al. 2017) and *Clostridium saccharobutylicum* (Huang et al. 2018) as well as in *Micrococcus luteus* (Surger et al. 2018) and *K.* *hansenii* ATCC 23,769 (Bimmer et al. 2022). Transformation of *K.* *hansenii* ATCC 53,582 was achieved by electroporation. Cells were grown in 100 mL complex medium to an OD_600_ of 0.8–1.0 and incubated with cellulase from *Trichoderma* *reesei* for 30 min to remove any cellulose. Then, the cultures were centrifuged for 10 min, 4 °C, and 3200 g, washed three times in ice-cold 1 mM HEPES, resuspended in 250 µL 1 mM HEPES and 50 µl 75% glycerol and stored in aliquots at − 80 °C. Plasmid DNA (100 ng) was added to an aliquot of the cells and the mix was incubated on ice for 10 min before applying the pulse. Electroporation of *K.* *hansenii* strains was done in 1-mm cuvettes using a Gene Pulser® II Electroporation System (Bio-Rad, Hercules, CA, USA) and applying the following settings: 25 kV/cm, 200 Ω, and 25 µF. For recovery, 900 µL of complex medium was added to the cells. After an incubation for 15 h at 30 °C and 230 rpm, the transformants were plated on selective complex medium plates containing 50 µg/mL kanamycin to select for plasmid integration. Clones positive for plasmid integration were plated for subsequent counter-selection on complex medium plates with reduced yeast extract and casamino acids and 120 µg/mL 5′-fluoro-cytosine. PCR amplification and Sanger sequencing (Eurofins Genomics, Ebersberg, Germany) was used to verify the constructed plasmids and deletion strains.

### Fourier transform infrared spectroscopy (FTIR) and X-ray crystallography (XRD)

Unless stated otherwise, cellulose pellicles were harvested after 6 days of static incubation, centrifuged for 10 min at 3200 g, and extensively washed in ddH_2_O to remove contaminants from the medium. Prior to FTIR spectroscopy and XRD measurements, the cellulose samples were dried over night at 50 °C until they reached a constant weight and the dry mass was measured. The characterization was performed using a frontier MIR spectrometer (L1280018) with an attenuated total reflection (ATR) diamond (PerkinElmer, Germany). All spectra were obtained from 32 scans with a resolution of 4 cm^−1^ and in the ATR mode using a wave number range from 400 to 4000 cm^−1^. For the determination of the crystallinity, X-ray diffractometry in Bragg–Brentano geometry (MiniFlex 600, Rigaku, Tokyo, Japan with D/teX Ultra, Copper Kα, Divergence Slits 0.625°, Soller Slits 2.5°) was used. The BC films were measured in the 2*θ* range between 3 and 60°, with scan steps of 0.02° at 5°/min. The crystallinity was calculated according to the Segal Peak Height method (Segal et al. 1959).

### Water absorption capacity (WAR)

The cellulose pellicles were extensively washed in ddH_2_O after cultivation in HS medium without agitation for 6 days. Water bound to the surface of the cellulose sheet was removed before determining the wet mass by horizontal draining for 30 min (Schrecker and Gostomski 2005). The dry mass was measured after overnight incubation at 50 °C, until a stable weight was reached. The WAR was calculated using Eq. (1) (Gullo et al. 2017):

1 $$WAR= \frac{{m}_{wet}-{m}_{dry}}{{m}_{wet}}\times 100\%$$

### Mechanical strength measurements—Young’s modulus

The mechanical strength of the different cellulose sheets synthesized by all *K.* *hansenii* strains that expressed BcsAB1 were measured on a modified Kieffer dough and gluten extensibility rig (Dunnewind et al. 2003). A special mount was constructed to ensure a thorough fixation of the wet BC during measurement (Figure S2). After 6 days of static cultivation, the pellicles were extensively washed in ddH_2_O before analysis. The samples were centrifuged for 10 min at 3200 g to remove excess water and ensure a comparable water content of all samples. The apparatus was calibrated by adjusting the measuring cell to 2 kg. For each biological sample, at least three technical replicates were measured. Therefore, each sample was cut into pieces of 75 mm × 10 mm and fixed in the mount. The Young’s modulus was calculated from the slope of the recorded force–displacement diagram as described by Dunnewind et al. (2003), and the cross-sectional area of each sample was considered during calculation. The thickness of the wet sheets was measured using a caliper.

### Scanning electron microscopy (SEM)

After 6 days of static cultivation, all samples were incubated overnight in 1 × PBS to remove remaining contaminants from the medium. The cellulose pellicles were dehydrated in an ascending EtOH series and stored in 100% EtOH until mounting and sputter-coating with platinum. As described before by Bimmer et al. (2022), the treatment of BC in EtOH is very gentle for putative EPSs and is preferable to acetone treatment prior to SEM analysis of BC from *Komagataeibacter* spp. The original orientation of the cellulose sheets was preserved during the whole procedure that the upper surface (facing the air during cultivation) was examined in SEM. The SEM micrographs were used to measure the diameter of the cellulose fibers produced by each strain.

### Transcription analysis: RNA extraction and RT-qPCR

The rapid and high-yield cellulose synthesis of *K.* *hansenii* ATCC 53,582 was a major hurdle for RNA extraction, since the cells were tightly embedded within the polysaccharide which could also bind the released RNA. To avoid any influence on the transcriptomic data, no treatment with cellulase was done after growth. The samples were taken after 3 days of cultivation in cellulase medium (see above), centrifuged for 10 min, 4 °C, and 3200 g, and immediately resuspended in TRIzol^TM^ Reagent (Thermo Fisher Scientific, Waltham, MA, USA) to extract RNA. Any gDNA was removed by DNase treatment (TURBO DNA-*free*^TM^ Kit, Thermo Fisher Scientific, Waltham, MA, USA). Absence of contaminating gDNA was confirmed by amplification of fragments of the ATP-synthase and the RNA-polymerase. Primers and procedures that were previously established for application in *K.* *hansenii* ATCC 23,769 (Bimmer et al. 2022) were also used in this study for *K.* *hansenii* ATCC 53,582. The tested PCR efficiency at 60 °C was kept within the limit defined in the general guidelines (Taylor et al. 2010). cDNA synthesis was done using the iScript™ Advanced cDNA Synthesis Kit (Bio-Rad, Hercules, CA, USA). The real-time PCR mix SsoAdvanced Universal SYBR Green Supermix (Bio-Rad, Hercules, CA, USA) was used for RT-qPCR and samples of 50 ng cDNA were measured in technical triplicates, as well as a no-template- and a no-RT-control. The analysis was run on a Bio-Rad CFX96™ cycler. After each measurement, a melting curve was made to ensure the specificity of the amplification products.

### Absolute quantification by real-time PCR

The expression level of the three CS (*bcsAB1*, *bcAB2*, and *bcsAB3*) was estimated by the absolute quantification method (Cusick et al. 2015; Whelan et al. 2003) after 3 days in a liquid culture containing 6 U/mL cellulase (from *T. reesei*, Sigma Aldrich, Steinheim, Germany). The reference gene *gyrA* was used as a control. A standard curve was prepared from a tenfold dilution series of genomic DNA spanning 1 × 10^1^ to 1 × 10^–5^ ng/µL (Hernández-Arriaga et al. 2019; Pfaffl 2012). For quantification, the threshold cycle (C_t_) was plotted against the logarithmic DNA target concentration. The corresponding DNA target concentration was calculated using Eq. (2) (Hernández-Arriaga et al. 2019):

2 $$DNA\;target\;concentration\;\left(copies\;\mu l^{-1}\right)=\frac{N_A\;\times\;DNA\;target\;amount\;\left(g\;\mu l^{-2}\right)}{M_W}$$

N_A_ = 6.02 *10^23^ (1/mol) (Avogadro constant).

M_W_ = DNA total length * 660 (g/mol).

### Protein analysis: sample preparation

Samples were taken after 3 days from cultures containing 6 U/mL cellulase (from *T. reesei*, Sigma Aldrich, Steinheim, Germany), centrifuged for 10 min, 4 °C, and 3200 g and washed twice in 1 × PBS to remove contaminating medium components. Addition of cellulase helped to prevent cellulose biofilm formation in samples for proteomic analysis. Cells were lysed in trifluoroacetic acid (TFA) for 5 min at 55 °C and subsequently neutralized by addition of 2 M Tris (pH = 8.2). A Bradford assay (Coomassie Plus Assay Kit, Thermo Fisher Scientific, Waltham, MA, USA) was performed for quantification of the total protein concentration. A total of 50 μg of protein per sample was reduced (8.3 mM TCEP) and carbamidomethylated (36.7 mM CAA) for 5 min at 95 °C. Digestion of the proteins was carried out by adding trypsin (proteomics grade, Roche) at a 1/50 enzyme/protein ratio (w/w) and incubation at 37 °C overnight. Digests were acidified by addition of 3% (v/v) formic acid (FA) and desalted using self-packed StageTips (five disks per micro-column, ø 1.5 mm, C18 material, 3 M Empore). The peptide eluates were dried to completeness and stored at − 80 °C. Before the LC–MS/MS measurement, all samples were freshly re-suspended in 12 µL 0.1% FA in HPLC grade water and 25 µg of total peptide amount were injected into the mass spectrometer per measurement. For each bacterial strain, four biological replicates were measured.

### Proteomic analysis: liquid chromatography–based tandem mass spectrometry (LC–MS/MS)

Peptides were analyzed on an UltiMate 3000 (micro-flow configuration) coupled to an Orbitrap Exploris 480 mass spectrometer (Thermo Fisher Scientific). Twenty-five micrograms of peptides was applied onto a commercially available Acclaim PepMap 100 C18 column (2 μm particle size, 1 mm ID × 150 mm, 100 Å pore size; Thermo Fisher Scientific) and separated using a two-stepped gradient: In the first step, a 50-min linear gradient ranging from 3 to 24% solvent B (0.1% FA, 3% DMSO in ACN) in solvent A (0.1% FA, 3% DMSO in HPLC grade water) at a flow rate of 50 μL/min was applied. In the second step, solvent B was further increased from 24 to 31% over a 10-min linear gradient. The mass spectrometer was operated in data-dependent acquisition (DDA) and positive ionization mode. MS1 full scans (360–1300 m/z) were acquired with a resolution of 60,000, a normalized AGC target value of 100%, and a maximum injection time of 50 ms. Peptide precursor selection for fragmentation was carried out using a fixed cycle time of 1.2 s. Only precursors with charge states from 2 to 6 were selected and dynamic exclusion of 30 s was enabled. Peptide fragmentation was performed using higher energy collision–induced dissociation (HCD) and a normalized collision energy of 28%. The precursor isolation window width of the quadrupole was set to 1.1 m/z. MS2 spectra were acquired with a resolution of 15,000, a fixed first mass of 100 m/z, a normalized automatic gain control (AGC) target value of 100%, and maximum injection time of 40 ms.

### Proteomic analysis: database searching

Peptide identification and quantification was performed using the software MaxQuant (version 1.6.3.4) (Tyanova et al. 2016) with its built-in search engine Andromeda (Cox et al. 2011). MS2 spectra were searched against all protein sequences obtained from NCBI, searching for “ATCC 53,582” and “ATCC 23,769,” respectively (2,811 proteins and 2,972 proteins, downloaded 22. Nov 2021), supplemented with common contaminants (built-in option in MaxQuant). The protein sequence database of *K.* *hansenii* ATCC 23,769 was manually completed by adding the protein sequences for BcsAB1, BcsC1, and BcsC2 from UniProt. Trypsin/P was specified as proteolytic enzyme. Precursor tolerance was set to 4.5 ppm, and fragment ion tolerance to 20 ppm. Results were adjusted to 1% false discovery rate (FDR) on peptide spectrum match level and protein level employing a target-decoy approach using reversed protein sequences. The minimal peptide length was defined as 7 amino acids; carbamidomethylated cysteine was set as fixed modification and oxidation of methionine and N-terminal protein acetylation as variable modifications. The match-between-run function was disabled. Estimations of protein mass fractions were carried out using the “intensity-based absolute quantification” (iBAQ) (Schwanhäusser et al. 2011) function implemented in MaxQuant. iBAQ values provide a quantification unit that is proportional to the absolute protein concentration in a given sample. By dividing the iBAQ value of a given protein through the summed iBAQ values of all proteins detected in a given sample, protein mass fractions were calculated.

### Motility assay

Pre-cultures of *K.* *hansenii* were grown on complex medium. New main cultures were inoculated to an OD_600 nm_ of 0.3 and incubated for 5 h at 30 °C. Five microliters from each culture was applied in the center of complex medium plates. For swimming analysis, the soft agar plates contained 0.3% agar. After 12 days at 30 °C, the diameter of each spot was measured using the measure tool from the GIMP software (open source).

### Statistics

All data was generated from at least three independent biological replicates. Data is represented as mean ± standard deviation (SD). Statistical differences between the wild type and the mutants were determined by Student’s *t*-test. A *p* value < 0.05 was considered statistically significant. Because some results could be considered extremely significant, the level of significance was specified by the number of asterisks and further determined in the result section as: * = *p* < 0.05; ** = *p* < 0.025; *** = *p* < 0.0001.

## Results

### Construction of* K. hansenii *ATCC 53,582 mutants

**Fig. 1:**
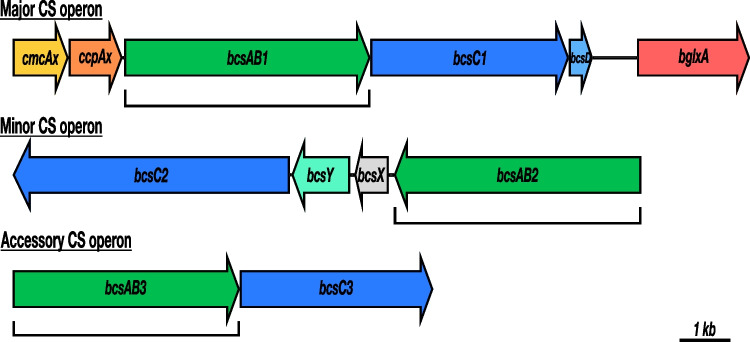
The genome of *K.* *hansenii* ATCC 53,582 (Florea et al. 2016) carries three putative cellulose synthase (CS) operons of different length and complexity. Each operon contains a CS gene (*bcsAB*) and a channel protein gene (*bcsC*), whereas the number of additional genes varies. *cmcAx,* carboxymethyl cellulase; *ccpAx,* cellulose complementing factor; *bcsD,* CS subunit D; *bglxA,* β-glucosidase; *bcsY,* acetyl transferase; *bcsX,* putative cellulose deacylase. The brackets mark the size and location of the in-frame deletions

*K. hansenii* ATCC 53,582 possess three CS genes in three operons of different complexity, partly together with genes for accessory proteins (Fig. 1). The *codBA* counter-selection system for acetic acid bacteria was established in our group by Kostner et al. (2013). Recently, it was successfully adopted to cellulose-producing acetic acid bacteria by Bimmer et al. (2022) being the method of our choice to construct in-frame deletions of the desired genes in the genome of the high-level cellulose-producing strain *K.* *hansenii* ATCC 53,582. Transformation was achieved by an adopted electroporation procedure for the different target strains (see above). It is worth to mention that the transformation efficiency was clearly enhanced after cellulase treatment (6 U/mL, 30 min) during preparation of the competent cells. See Table 1 for a list of all mutants generated within this study. Since, it was not possible to generate a double deletion of the diguanylate cyclases *dgcA* and *dgcB*, we assume that the presence of at least one of these DGCs is essential. A analogous observation was previously reported for *K.* *hansenii* ATCC 23,769 (Bimmer et al. 2022).

### Characterization of cellulose formation

#### Phenotypical aspects

**Fig. 2:**
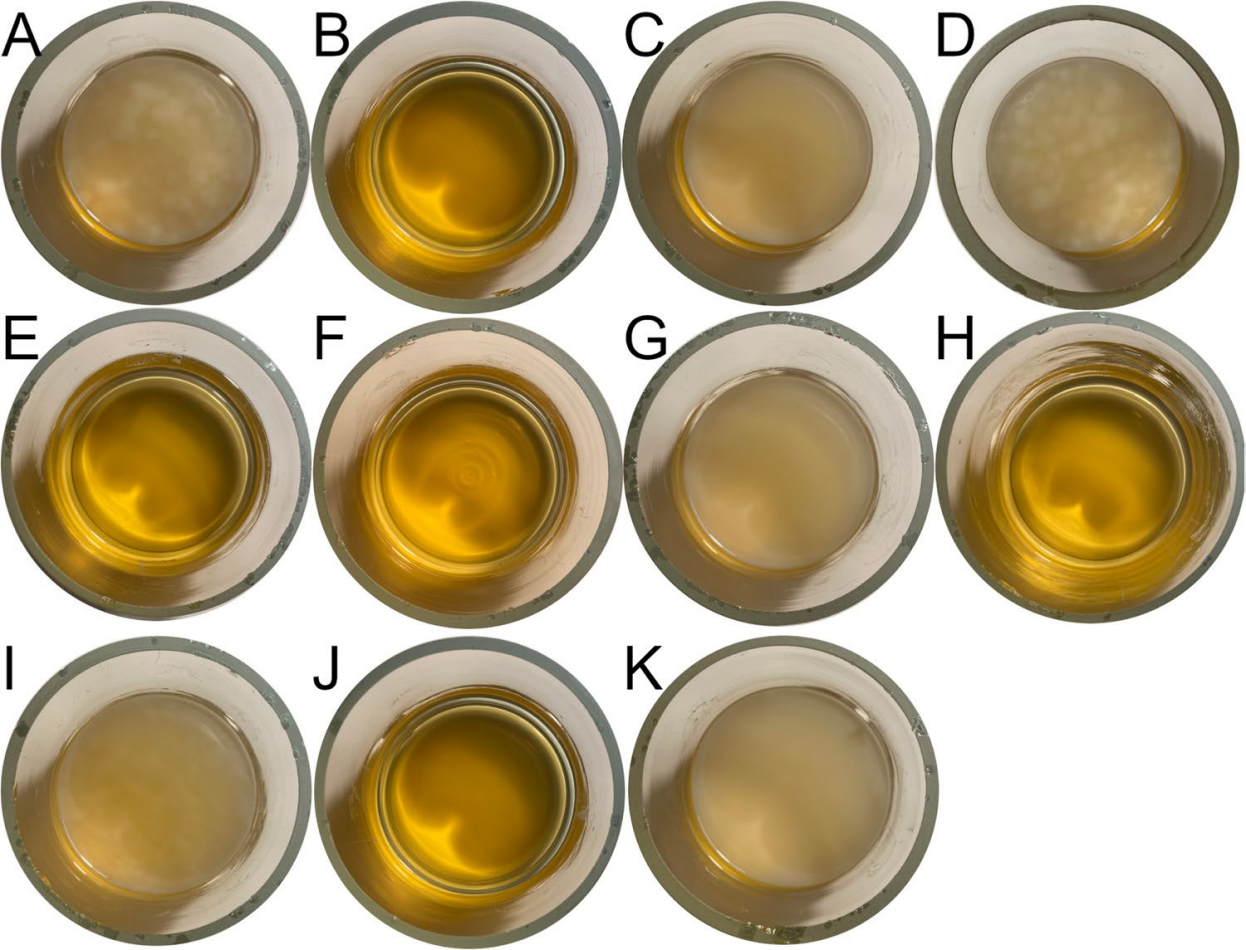
Phenotypes of BC synthesis of *K.* *hansenii* ATCC 53,582 and its CS clean deletion strains after 14 days of cultivation in HS medium without agitation. **A** WT; **B** Δ*bcsAB1*, no BC formed after deletion of the main CS; **C** Δ*bcsAB2*, no effect on the phenotype of BC; **D**: Δ*bcsAB3*, no effect on the phenotype of BC; **E** Δ*bcsAB1* Δ*bcsAB2*, no BC formed after simultaneous deletion of the main CS and *bcsAB2*; **F** Δ*bcsAB1* Δ*bcsAB3*, no BC formed after simultaneous deletion of the main CS and *bcsAB3*; **G** Δ*bcsAB2* Δ*bcsAB3*, no effect on the phenotype of BC; **H** Δ*bcsAB1* Δ*bcsAB2* Δ*bcsAB3,* no BC formed after simultaneous deletion of the three CS; **I** Δ*dgcA*, no effect on the phenotype of BC; **J** Δ*dgcB*, no BC formed after deletion of *dgcB*; **K** Δ*bcsY*, no effect on the phenotype of BC

Differences in surface cellulose formation of the *K.* *hansenii* ATCC 53,582 CS markerless deletion strains generated in this work were easily observable in static and in shaking cultures. For an improved visualization of the different phenotypes, the images were taken after 14 days of cultivation on glucose without agitation (Fig. 2). The contribution of *bcsAB1* to the formation of large amounts of cellulose is apparent. Strains expressing *bcsAB1* in any combination with or without expression of the additional CS genes *bcsAB2* and/or *bcsAB3* formed a thick cellulose layer at the medium surface (Fig. 2A, C, D, G). In contrast, any strain deficient in the major CS, BcsAB1, therefore only expressing BcsAB2 and/or BcsAB3 was not able to produce any kind of EPS macroscopically visible at the surface (Fig. 2B, E, F, H). Furthermore, in these cultures, the growth medium also showed no turbidity that would indicate strong growth without of EPS formation after static cultivation of these strains.

Besides the chromosomal clean deletions of the CS genes, the contribution of the acetyl transferase *bcsY* (encoded close to *bcsAB2*) and the regulatory DGCs (*dgcA*, *dgcB*) were assessed by in-frame deletions of the respective genes. The deletion of *bcsY* showed no altered phenotype (Fig. 2K). No difference was visible compared to the phenotype after deletion of the nearby located CS *bcsAB2* (Fig. 2C). The deletion of *dgcA* generated a slightly weaker and less compact pellicle compared to the wild type (Fig. 2I), whereas the deletion of *dgcB* resulted in a phenotype with no cellulose synthesis (Fig. 2J).

#### Dry mass

Due to the rapid production of a large amount of cellulose by *K.* *hansenii* ATCC 53,582, a modified cultivation method was used, based on a reduced inoculum size and a shorter incubation time. This procedure was different from the method previously used for the lower yield strain *K.* *hansenii* ATCC 23,769 (Bimmer et al. 2022). This new method was used as a fast and easy tool for evaluation of cellulose synthesis of the generated mutants (the difference in cellulose yield of the two strains ATCC 23,769 and ATCC 53,582 is nicely depicted in Fig. S1). It must be pointed out, however, that the procedure has not been optimized to maximize cellulose production. The effect of the different deletions was quantified by measurement of the dry mass of extensively washed cellulose sheets after 6 days of growth on glucose. To this end, the BC samples were dried to a constant weight in an oven at 50 °C. For *K.* *hansenii* deletion strains that form no pellicle (deletion of *bcsAB1* or *dgcB*), no dry mass could be measured. The wild type produced an amount of 118.5 ± 20.2 mg/L of BC when it was grown for 6 days on HS medium in a beaker with 10-cm surface diameter. The deletion of BcsAB2 (99.1 ± 15.8 mg/L) and BcsAB3 (116.2 ± 22.0 mg/L) as single as well as double deletions (106.1 ± 21.6 mg/L) and the deletion of BcsY (117.3 ± 17.9 mg/L) had no significant effect on overall cellulose production. However, the single deletion of BcsAB2 seems to show a slight tendency of a reduced BC dry mass. These results indicate that the polysaccharide synthesis by BcsAB2 and BcsAB3 is low in *K.* *hansenii* ATCC 53,582, and microfibrils generated by BcsAB1 dominate the cellulose formed by this strain. *K.* *hansenii* Δ*dgcA* shows a significantly reduced BC mass compared to the wild type (52.4 ± 16.8 mg/L), whereas *K.* *hansenii* Δ*dgcB* shows no cellulose formation at all. Therefore, we identified *dgcB* as key regulatory DGC that is essential for cellulose synthesis in *K.* *hansenii* ATCC 53,582, whereas the deletion of *dgcA* resulted in a 58% decline of cellulose formation.

#### Scanning electron microscopy

**Fig. 3:**
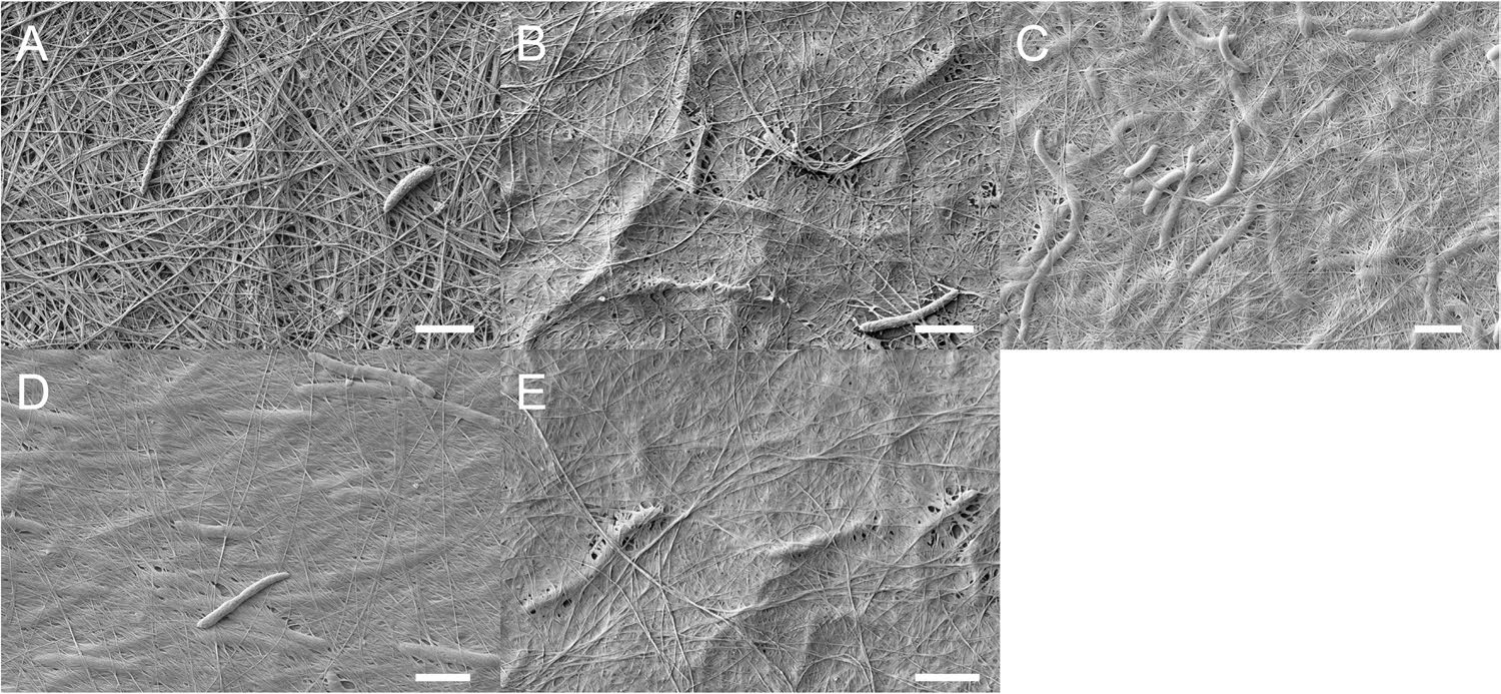
SEM micrographs of BC synthesized by *K.* *hansenii* ATCC 53,582 and its CS deletion mutants. The samples were harvested after 6 days of static cultivation and dehydrated in an ascending EtOH series. Consequences of the deletions of the additional CS *bcsAB2* and/or *bcsAB3* on the cellulose network. All mutants seem to show a tighter network of cellulose fibers with smaller diameter of the microfibrils. **A** WT; **B** Δ*bcsAB2*; **C** Δ*bcsAB3*; **D** Δ*bcsAB2* Δ*bcsAB3*; **E** Δ*bcsY*. The scale bar represents a size of 2 µm

Macroscopic inspection of cellulose formation in cultures of the CS mutant strains did not indicate a clear contribution of the minor and the accessory CS on cellulose biofilm production. Therefore, the microstructure was analyzed using SEM. Our previous study of *K.* *hansenii* ATCC 23,769 (Bimmer et al. 2022) revealed the importance of an appropriate dehydration procedure to preserve amorphous EPSs and to maintain the native arrangement of the microfibrils within the network. Therefore, the samples were dehydrated without acetone in an ascending EtOH series before platinum-sputtering and mounting. A network of many cellulose fibrils and only a few cells on the upper surface of the cellulose pellicle are easily distinguishable in SEM micrographs of *K.* *hansenii* ATCC 53,582 (Fig. 3A). Various interconnected planes of cellulose fibers and certain loose regions within the network can be observed. However, the single deletion of *bcsAB2* and *bcsAB3* and their double deletion showed a modification of the SEM micrographs (Fig. 3B, C, D). On the one hand, the BC formed by these mutants still forms a network of cellulose fibers. On the other hand, the fibers seem to be finer and more densely packed; i.e., each fiber is in closer contact with other fibers, resulting in a tighter network. Although no additional EPS synthesized by BcsAB2 or BcsAB3 was observed, it is obvious that the products of these additional CSs alter the morphology of the cellulose fibers—visible by changes of the overall organization of the network.

After assessing the effects of the different CS genes on BC network formation, the effects of the remaining clean deletions on the assembly of the fibril scaffold were determined. The deletion of *dgcA* resulted in a SEM phenotype that is comparable to the wild-type situation. The deletion of the acetyl transferase *bcsY* (Fig. 3E), a gene of the *bcsAB2* operon, resulted in an altered phenotype as observed by electron microscopy. The network seems to be less loose and each of the observable fibers seems to be in a tighter contact to its neighboring fibers, while the resolution of the spatial depth seems to be reduced. These results are similar to the observations mentioned above for deletion of *bcsAB2*.

#### Characterization of the cellulose fibers

The SEM micrographs revealed clear differences in the appearance of the thickness of the cellulose fibers formed by the different mutants when compared to the wild-type phenotype. To assess the contribution of the minor and accessory cellulose synthase as well as of the acetyltransferase on fiber assembly, the diameter of some of the cellulose fibers were measured in the SEM images using the integrated measuring tool. The measurements confirmed the significant reduction in fiber diameter after the deletions. While the average diameter of the *K.* *hansenii* wild type is 160 nm, the fiber size was significantly reduced after deletion of the minor (78 nm) and the accessory cellulose synthase (103 nm). The strongest reduction was observed after simultaneous deletion of *bcsAB2* and *bcsAB3* (67 nm) as well as after deletion of *bcsY* (67 nm) (Table 3).

**Table 3:** Physicochemical parameters of BC generated from *K.* *hansenii* strains

Strain	CI [%] ± SD	Young’s modulus [MPa] ± SD	WAR [%] ± SD	Diameter [nm] ± SD
*K. hansenii* ATCC 53,582	77.20 ± 2.85	145.2 ± 12.4	99.5 ± 0.1	160 ± 40
*K. hansenii* Δ*bcsAB2*	76.25 ± 3.09	284.1 ± 36.7 ***	99.6 ± 0.1	78 ± 22 **
*K. hansenii* Δ*bcsAB3*	74.88 ± 1.91 **	119.9 ± 13.8 *	99.5 ± 0.1	103 ± 2 **
*K. hansenii* Δ*bcsAB2* Δ*bcsAB3*	74.55 ± 2.14 *	272.5 ± 14.0 ***	99.3 ± 0.0 **	67 ± 7 **
*K. hansenii* Δ*bcsY*	74.72 ± 2.93 *	235.1 ± 0.8 ***	99.4 ± 0.0	67 ± 14 ***
*K. hansenii* Δ*dgcA*	75.39 ± 3.41	230.2 ± 67.1 *	99.7 ± 0.0 **	–

**Fig. 4:**
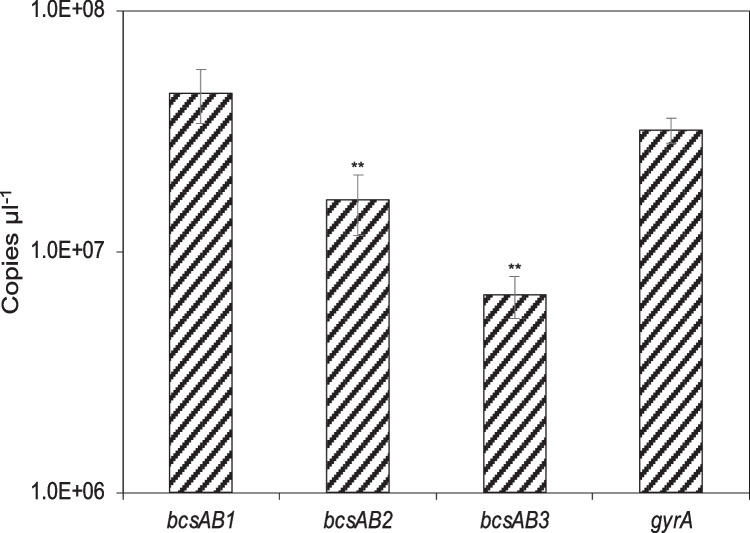
Transcription analysis of *K.* *hansenii* ATCC 53,582 CS genes by RT-qPCR. Absolute quantification of CS mRNA levels was done after 3 days of cultivation in cultures containing cellulase. CS transcription was compared to the reference gene *gyrA*. [mean ± SD; *n* = 4]

The crystallinity index (CI) was determined using dried cellulose pellicles after cultivation for 6 days at 25 °C on glucose without agitation. The analysis of the peaks was done by Segal Peak Height calculation [mean ± SD; *n* = 5 ≤ 11]. The Young’s modulus values were determined with wet BC after 6 days of cultivation without agitation. The mechanical strength was calculated from the slope of the linear part of the stress–strain diagram after vertical extension [mean ± SD; *n* = 3 ≤ 5]. The water absorption ratio (WAR) of BC after 6 days of static cultivation on glucose medium was analyzed after oven drying at 50 °C until stable weight was reached [mean ± SD; *n* = 3]. The diameters were determined on the basis of the SEM micrographs shown in Fig. 4 using the integrated measuring tool of the SEM analysis software (*n* = 4–5)

#### Transcription analysis by RT-qPCR

To understand the differences in quantitative cellulose formation and the mentioned modifying effects of the three CS genes, *bcsAB1, bcsAB2*, *and bcaAB3*, their mRNA levels were measured. Transcription analysis was performed in cellulose-producing cultures. Since the high amounts of released cellulose interfered with RNA extraction, *K.* *hansenii* ATCC 53,582 was grown in HS medium in the presence of cellulase (6U/mL, from *Trichoderma reesei*) and in shaking cultures (60 rpm; 30 °C) for 3 days. In parallel, a culture without addition of cellulase was grown to confirm cellulose formation under these conditions. The absolute quantification after RT-qPCR analysis of the extracted RNA revealed transcription of each of the three cellulose synthase genes as shown in Fig. 4. As expected, the transcription of the major CS *bcsAB1* is the highest (4.54 × 10^7^ copies/µl), confirming its importance for rapid and high-level synthesis of cellulose. The abundance of the transcript of the minor CS *bcsAB2* was significantly (about 2.8-fold) lower (1.63 × 10^7^ copies/µl) compared to the mRNA level of *bcsAB1*, which agrees with macroscopic observations and SEM micrographs. The mRNA level of *bcsAB3* was even lower (6.61 × 10^6^ copies/µl) compared to the other two CS genes, being about 8.4-fold lower than *bcsAB1* and 2.5-fold lower than *bcsAB2*. An analogous transcription of these three CS genes was also seen after 6 days of static cultivation (data not shown).

#### Proteomics

Mass spectrometry–based proteomics was used to investigate the protein concentrations of the CSs and the accessory proteins encoded in the major CS operon. *K.* *hansenii* ATCC 53,582 WT and its Δ*bcsAB2* Δ*bcsAB3* double mutant, expressing only BcsAB1, were grown and processed analogously to the procedure for transcriptomic analysis in the medium containing cellulase to prevent the formation of cellulose.

**Fig. 5:**
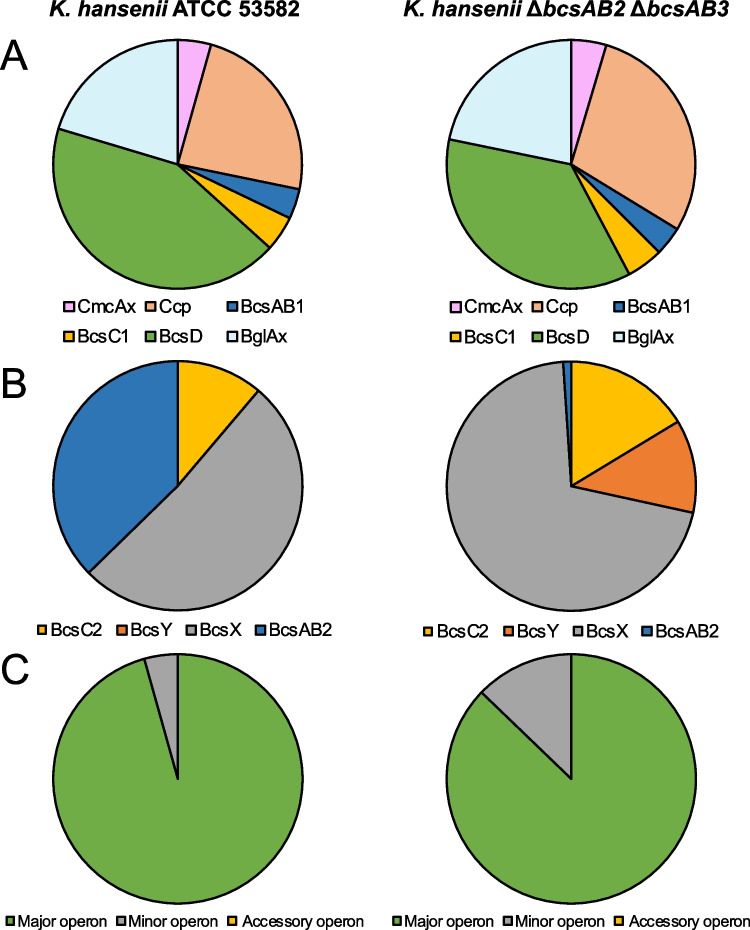
Relative levels of expression of proteins encoded in the CS operons in *K.* *hansenii* ATCC 53,582 WT (left column) and *K.* *hansenii* Δ*bcsAB2* Δ*bcsAB3* (right column). **A** Relative abundance of the proteins associated with BcsAB1—the deletions of the additional CS have no strong effect on the expression of the proteins encoded with BcsAB1. **B** Relative abundance of the proteins associated with BcsAB2—the deletions resulted in an increase of the relative expression of BcsY and BcsX. **C** The main CS shows the highest relative abundance of the proteins of the three CS operons in *K.* *hansenii* ATCC 53,582 and in *K.* *hansenii* Δ*bcsAB2* Δ*bcsAB3*. [mean; *n* = 4]

The proteomic analysis confirmed the expression of BcsAB1 in equivalent levels for the wild type and the CS double deletion strain (Fig. 5A). In contrast, the abundance of BcsAB2 was significantly lower than BcsAB1 in the wild type, confirming the transcriptomic data, and almost absent in the double deletion strain, as expected. The detection of BcsAB2 in the *bcsAB2*-*bcsAB3*-deletion mutant was limited to the identification of a single peptide, likely indicating a false positive hit (Fig. 5B). In contrast to the transcription data, not a single peptide from BcsAB3 could be detected by proteomics (Fig. 5C). Like BcsAB3, also the operon partner protein BcsC3 was not detected in the proteomic data. This hints to the fact that BcsAB3 as well as BcsC3 are either not expressed, or expressed with low protein concentrations (at least below the detection limit of the mass spectrometer).

In total 2030 proteins were detected within the proteomics analysis. Compared to the whole proteome, the CSs showed an average level of expression, while the accessory proteins BglxA, CcpAx, and CmcAx, as well as BcsD, were slightly more frequent than the average proteins (Fig. S3).

Since the first CS operon is described as an operon of *bcsABCD*-type (Römling and Galperin 2015; Wong et al. 1990), a comparable expression of each protein of the operon was assumed. The clearly higher abundance of BcsD (7.6-fold of BcsAB1) suggests its significant involvement in the CS complex for assembly of the growing cellulose fiber (Fig. 5A). Another interesting aspect of the proteomic data was the analysis of the accessory proteins encoded close to BcsAB1 that showed a significantly high expression level and contributed to almost half of the proteins encoded close to the major CS. The accessory proteins CcpAx (6.1-fold/5.2-fold) and BglxA (5.2-fold/4.4-fold) were also present in high abundance, relative to BcsAB1 and BcsC1. After simultaneous deletion of BcsAB2 and BcsAB3, in the strain expressing only BcsAB1, no changes regarding the expression of CmcAx, CcpAx, and BglxA were observed (Fig. 5A).

On the other hand, the acetyl transferase (BcsY), which is the enzyme we suggested for modification of BC in *K.* *hansenii* ATCC 23,769 (Bimmer et al. 2022), was only detected after deletion of BcsAB2, while the expression of BcsX and BcsC2, the other proteins of the minor operon, was strongly increased in the deletion mutant (4.3-fold/4.0-fold) (Fig. 5B). By analyzing all proteins contributing to fiber assembly, the outstanding contribution of the major operon 1 on cellulose formation in *K.* *hansenii* ATCC 53,582 was demonstrated (Fig. 5C). Moreover, in terms of protein abundance, the proteomic data indicate a merely small contribution of the minor CS, BcsAB2, and the accessory CS, BcsAB3, since over 80% of the detected proteins originated from the major operon.

### Physicochemical characterization of cellulose

#### FTIR and crystallinity

The in-frame deletion of the additional CS genes *bcsAB2* and *bcsAB3* resulted in clearly different cellulose appearance according to the SEM micrographs, which indicated their contribution to fiber assembly. The dried cellulose samples prepared from the different CS mutants were analyzed by Fourier transform infrared spectroscopy (FTIR) to detect chemical differences. However, according to the recorded spectra, all samples were identified as microcrystalline cellulose (data not shown). Moreover, no hints for acetyl groups or any other modification that may explain the differences in fiber formation were detected. X-ray diffractometry was applied for further characterization to assess the crystallinity index (CI) by calculation according to the Segal Peak Height (Segal et al. 1959) method (Table 3). In contrast to the formation of amorphous cellulose species as described for *K.* *hansenii* ATCC 23,769 (Bimmer et al. 2022), the cellulose synthesized by *K.* *hansenii* ATCC 53,582 showed a distinct crystallinity. Although quantification of the different samples showed no significant change after in-frame deletion of the additional CS genes, the deletion of *bcsAB3* decreased the CI significantly to 74.88%, compared to 77.20% of the wild type. Moreover, only a slight decrease was measurable after deletion of *bcsAB2* (76.25%). However, the double deletion of *bcsAB2* and *bcsAB3* resulted in a significant reduction of its crystallinity (74.55%). Despite the inability to detect acetylation of the cellulose, the significant reduction of the crystallinity after deletion of the proposed acetyl transferase gene *bcsY* (74.72%)—located between *bcsAB2* and *bcsC2*—is intriguing. Together with the reduction of the crystallinity observed for the *bcsAB2-* and *bcsAB3-*deficient mutants, these results demonstrate the modifying influence of the additional CS gene clusters on the cellulose fibers formed by BcsAB1. The diffractograms revealed that the formed BC was mainly cellulose I⍺ (French 2014).

#### Mechanical strength of BC—Young’s modulus

BC is a versatile and sustainable biomaterial used in many biotechnological, biomedical, or food technological applications. Especially its high mechanical strength makes BC an interesting material for the construction of 3D scaffolds for tissue engineering. The effect of the clean deletions in the CS gene clusters on the mechanical properties of BC was analyzed by measuring the vertical extension of a cellulose sheet and calculation of the Young’s modulus as described in the “Materials and methods” section. Tensile strength was measured after 6 days of cultivation on glucose without agitation, using material from extensively washed pellicles. Cells integrated within the BC network were not removed during sample preparation to avoid any distortion or modification induced by a harsh treatment. The calculated Young’s modulus of the wild type was 145.2 ± 12.4 MPa (Table 3). Any deletion within the minor CS operon, i.e., the deletion of *bcsAB2* as well as *bcsY*, increased the mechanical strength of the cellulose significantly (*K.* *hansenii* Δ*bcsAB2*: 284.2 ± 36.7 MPa; *K. hansenii* Δ*bcsY*: 235.1 ± 0.8 MPa), which may support the idea that these genes are involved in cellulose fiber modification. The cellulose generated by the double mutant *K.* *hansenii* Δ*bcsAB2* Δ*bcsAB3* also revealed a significantly increased value of the Young’s modulus (272.5 ± 14.0 MPa), whereas the single Δ*bcsAB3* mutant did not result in an increase, but rather a slight decrease compared to the wild type (119.9 ± 13.8 MPa). *K.* *hansenii* Δ*dgcA* generated cellulose with an apparently high variability of its mechanical strength (relatively large variation among assay repetitions), but on average resulted in an increased mechanical strength (230.2 ± 67.1 MPa) compared to the wild type.

#### Water content

The effect of the additional CSs on the fiber assembly was already described by the change of the physicochemical properties of BC. Differences in the BC fine structure may also indicate variations in the water holding capacity of the pellicle. After 6 days of static cultivation, the water absorption ratio (WAR) was higher than 99% for all tested strains (Table 3). However, the deletion of *dgcA* significantly enhanced the WAR of the cellulose compared to the wild type (99.7% vs. 99.5%). On the other hand, the WAR after double deletion of *bcsAB2* and *bcsAB3* was significantly lower (99.3%) than found for the wild-type BC. The WAR of the other mutants remained unchanged when compared to the wild type.

#### Motility assay

Cellulose formation as well as cell motility is regulated via regulatory binding of the intracellular second messenger cyclic-di-GMP (Ross et al. 1987). To understand the physiologic roles of the two diguanylate cyclases (DGCs) encoded in the genome of *K. hansenii* for each of those processes, the consequences of deletion of *dgcA* and *dgcB* on cell motility were monitored using a motility assay. The assay measuring the swimming activity was accomplished on soft agar plates (0.3% agarose). It revealed a significant decrease of the relative diameter of 35% and 40% for each of the respective DGC deletion strains compared to the wild type after 12 days. The consequences of the deletion of *dgcA* and *dgcB* on cellulose formation were already mentioned above (see Fig. 2).

## Discussion

### Cellulose formation by CS and DGC mutants

A comprehensive understanding of the genetics of BC synthesis is necessary to define targets to engineer its properties. This strategy could be used to optimize the cellulose yield or tune its characteristics to the product needs in biotechnology, biomedicine, and food technology. The presence of three different CS operons (Umeda et al. 1999; Wong et al. 1990) in cellulose-producing *K. hansenii* became evident after elucidation of the whole genome sequence (Florea et al. 2016). This was followed by different gene disruption approaches—such as transposon or chemical mutagenesis—in several cellulose-producing acetic acid bacteria strains (Buldum and Mantalaris 2021). However, these studies are associated with polar effects because they lack chromosomal clean deletions (Ryngajłło et al. 2020). The *codBA* counter-selection system for genome editing of a high-yield cellulose-producing acetic acid bacterium established in this study now enables the construction and characterization of clean, markerless deletions in the genome. Our group has previously demonstrated that this deletion system can be broadly used for various Gram-positive and Gram-negative bacteria from different genera (Huang et al. 2018; Kostner et al. 2013, 2017; Surger et al. 2018) and was recently adapted to *K.* *hansenii* ATCC 23,769 (Bimmer et al. 2022). The genes for BC formation in strain ATCC 23,769, which produces cellulose at a lower yield, and in the high-yield cellulose-producing strain *K.* *hansenii* ATCC 53,582 show a homologous organization in three independent chromosomal regions containing the CS operon (Florea et al. 2016; Iyer et al. 2010). But the yield and production rate of BC in the latter strain is much higher and faster, and therefore of particular industrial relevance.

In this study, we report that strain ATCC 53,582 forms BC consisting mainly of cellulose fibrils synthesized by the major CS BcsAB1. The products of BcsAB2 and BcsAB3 are formed in a significantly lower amount than previously reported for *K.* *hansenii* ATCC 23,763 (Bimmer et al. 2022). Unlike the lower-yield cellulose-producing strain ATCC 23,769, no patches of EPS were formed on the medium surface after static cultivation of *K.* *hansenii* ATCC 53,582 with a deleted major CS operon (Fig. 2).

This study for the first time links the expression of DgcB in *K.* *hansenii* ATCC 53,582 to the regulation of cellulose synthesis (Fig. 2). This result indicates an even higher contribution of DgcB on activation of BcsA than previously described for *K.* *hansenii* ATCC 23,769, where a fragmented biofilm was produced after deletion of *dgcB* (Bimmer et al. 2022). Other studies found no strong reduction of biofilm formation in response to DGC mutation in cellulose-producing acetic acid bacteria. The disruption of the DGC contributing to the highest portion of cyclic-di-GMP in *Acetobacter xylinum* induced only a slight reduction in biofilm formation (Bae et al. 2004; Tal et al. 1998). In the work reported by Tal et al. (1998), the disruption of *cdg1* or *cdg2,* which are located in a homologous genome region, led to different results. These differences in the phenotype may be directly linked to the gene disruption technique used. While we constructed markerless chromosomal in-frame deletions, the previously reported gene disruptions were created by insertion of an antibiotic cassette, which may result in polar effects.

### Microstructure of the BC

**Fig. 6:**
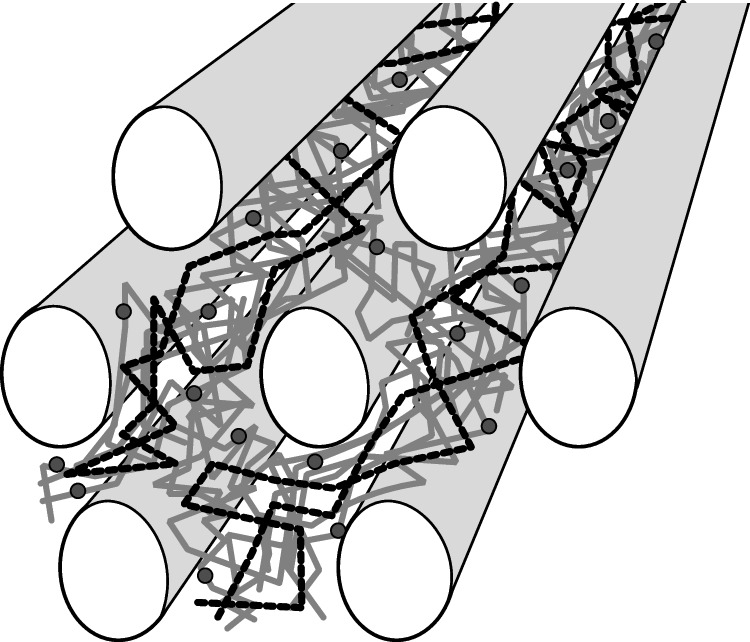
Proposed scheme of the structure of a BC fiber in *K.* *hansenii* ATCC 53,582. Several cellulose fibrils synthesized by BcsAB1 are stuck together by the polymers released as products of BcsAB2 and BcsAB3, respectively. Light gray tubes = cellulose fibers (product of BcsAB1), gray lines = product of BcsAB2, gray dots = possible modifications of the nfEPS from BcsAB2 induced by BcsY and black dashed lines = product of BcsAB3

Microstructural analysis using SEM complemented the macroscopic picture of the cellulose pellicles. Since no polymer was observed on the surface of cultures after single expression of either *bcsAB2* or *bcsAB3*, SEM of the BC formed by mutants lacking the minor and/or accessory CS genes was carried out to study their possible contributions. The micrographs of the wild type are dominated by well-defined cellulose fibers and also significant voids within the cellulose network (Fig. 3). The synthesis of these cellulose macrofibrils can explicitly be attributed to the expression of BcsAB1. No additional EPS that could be attributed to the presence of BcsAB2 or BcsAB3 was visible in SEM micrographs of the wild type or of the mutants. These results are in contrast to observations made with the closely related lower-yield cellulose-producing strain *K.* *hansenii* ATCC 23,769, where an nfEPS (non-fibrous EPS) was observed by SEM and was linked to the presence of BcsAB2 and BcsAB3 (Bimmer et al. 2022). Nevertheless, we suggest a strong contribution of BcsAB2 and BcsAB3 to the formation of thick cellulose fibers by *K.* *hansenii* ATCC 53,582 nicely visible in the SEM micrograph of BC formed by the wild type. We assume that the quantity of the EPS was too low to give rise to visible patches of cellulose on the culture surface after static cultivation of single-expression mutants of *bcsAB2* and *bcsAB3* or after deletion of the major CS *bcsAB1*—unlike the analogous mutant of the lower-producing strain ATCC 23,769. On the other hand, SEM analysis of the ATCC 53,582 wild type and its CS mutants indicates that BcsAB2 and BcsAB3 clearly affect fiber formation. In the wild type, the nfEPS produced by the minor and accessory CS may be completely absorbed by the fibers formed by the major operon, resulting in a kind of “binder effect” leading to the formation of thicker fibers. We draw a scheme to illustrate our idea for the putative fiber assembly (Fig. 6). The microcrystalline fibers formed by BcsAB1 are embedded in and connected by the nfEPS products of BcsAB2 and BcsAB3.

Unlike previously described for *K.* *hansenii* ATCC 23,769, where the nfEPSs formed by BcsAB2 and BcsAB3 were not absorbed completely by the cellulose fibers of BcsAB1, but were visible as polymer matrix embedding the cells and fibers (Bimmer et al. 2022). The observed apparent absorption of nfEPS into the fibers in *K.* *hansenii* ATCC 53,583 could affect the mechanical properties of the cellulose-fiber network, allowing an improved flexibility. The lack of the “binder-like substance” after deletion of *bcsAB2* and *bcsAB3* reduced the fiber thickness resulting in more felted cellulose fibrils and may affect the ability of the fibrils to slide against each other. Based on our results and the interpretation just formulated, a functional similarity of bacterial nfEPS with the function of hemicelluloses in plant lignocellulose can be proposed. While hemicelluloses regulate the aggregation of cellulose fibers during formation of the primary cell wall in plants (Atalla et al. 1993; Scheller and Ulvskov 2010), the observed effects of the products from BcsAB2 and BcsAB3 may modify BC in an analogous way. Conversely, this could increase the porosity of the fiber network affecting the water holding capacity as well as the tensile strength. Biotechnological applications of BC as filter membrane or packaging material in food packaging could benefit from these observations. Since certain filter membranes are characterized by the diameter of the filter pores (Fillat et al. 2018; Galdino et al. 2020; Lehtonen et al. 2021), adjustment of the relative expression levels of the different CSs may represent a new target to rationally design the properties of BC filter membranes.

### Transcription analysis of CS expression

Apart from bulk BC fiber synthesis by the major CS BcsAB1, fiber assembly is affected by the minor and accessory CS. All three CSs appear to play a key role in natural BC formation by *K.* *hansenii* ATCC 53,582. Therefore, it was of special interest to investigate the transcription levels of *bcsAB1*, *bcsAB2*, and *bcsAB3*. Previously, only a few studies have focused on the transcription analysis of CSs in *Komagataeibacter* species. These studies dealt with other aspects that influence the transcription pattern of the CSs: e.g., phytohormone treatment (Augimeri and Strap 2015), analysis of accessory proteins (Deng et al. 2013; Kawano et al. 2008), or strain characterization of a related *Komagataeibacter* strain (Hernández-Arriaga et al. 2019). RNA extraction from *K.* *hansenii* ATCC 53,582 cultures under cellulose-producing conditions was difficult. Since it was not possible to extract high-quality RNA after static cultivation, we used gently agitated cultures supplemented with cellulase, a procedure previously used for transcription analysis after phytohormone treatment of this strain (Augimeri and Strap 2015). As cellulose can cover cells and impede cell lysis or adsorb released RNA, cellulase treatment was used to obtain a culture free of cellulose. The cellulase treatment was not done after growth, since the necessary incubation would have altered the transcriptome (Moffitt et al. 2016). The mRNA level of *bcsAB1* was significantly higher than that of *bcsAB2* and *bcsAB3* (Fig. 4) supporting the characterization of *bcsAB1* as the major CS gene that forms the bulk of the cellulose fibers. However, the comparatively high transcription level of *bcsAB2* supports a significant contribution to cellulose network formation. This contrasts the low transcription level of *bcsAB3* (14.6% of *bcsAB1* mRNA) which may indicate a low overall contribution of this gene. A study in *K.* *medellinensis* that dealt with the analysis of the four CS genes of this strain revealed a comparable situation. Two *bcs* clusters (*bcs1* and *bcs4*) were highly transcribed, while the other two clusters (*bcs2* and *bcs3*) showed significantly lower transcription (Hernández-Arriaga et al. 2019). Comparison of the CSs by BLASTp revealed homologies of Bcs1 and BcsAB1, Bcs4 and BcsAB2, and Bcs3 and BcsAB3. No corresponding protein for *K.* *medellinensis* Bcs2 was identified in *K hansenii* ATCC 53,582. The genetic organization of the clusters show a comparable decrease in complexity and number of accessory proteins from Bcs1 to Bcs4. Based on these homologies, the importance of CSs homologous to BcsAB1 and BcsAB2 may be common in acetic acid bacteria that contain more than one CS. However, a detailed characterization of the products of *bcs2*, *bcs3*, and *bcs4* in *K.* *medellinensis* is not available.

### Abundances of proteins involved in BC synthesis

Since transcription analysis of the CS genes revealed a significant difference in mRNA levels of the major, minor, and accessory CSs, proteomic analysis can enhance the understanding of the fundamentals of their different phenotypical contributions. The high portion of the proteins encoded in the major CS operon within all proteins linked to cellulose synthesis proves the importance of this operon, but also suggests that the accessory proteins play important roles. The major CS operon of *K.* *hansenii* ATCC 53,582 is characterized by its *bcsABCD* structure and the accompanying genes *cmcAx*, *ccpAx* and *bglxA* (Florea et al. 2016; Römling and Galperin 2015; Wong et al. 1990). The remarkably high abundance of BcsD in comparison to BcsAB1 indicates an important role of this protein for cellulose production (Fig. 5). BcsD is a small protein of 17 kDa localized in the periplasm. It forms a cylindrical octamer with a central pore, which facilitates the formation of glucan chain bundles (Hu et al. 2010). Reduced cellulose production after disruption of *bcsD* suggests a contribution to cellulose crystallization (Mehta et al. 2015; Saxena et al. 1994). Studies of a fluorescent fusion to Ccp (resembling CcpAx in Fig. 1) identified its localization along the longitudinal axis of the cell and its co-occurrence with BcsD (Sunagawa et al. 2013). These results are in accordance with a recently identified novel cytoskeletal element in the genus *Komagataeibacter* (*Gluconacetobacter*)—the cortical belt. The cortical belt is near the cytoplasmic membrane and underlies the regions where cellulose emerges from the cells and may have a function in aligning the enzyme complexes involved in cellulose fiber formation (Nicolas et al. 2021). Our proteomic analysis also revealed that BglxA, a periplasmatic beta-glucosidase (Römling and Galperin 2015; Tajima et al. 2001; Tonouchi et al. 1997), represented almost one-fourth of the proteins expressed by the major operon, highlighting the importance of this protein for the assembly of cellulose fibers. Thus, the proteomic analysis corroborates the idea that the mentioned accessory proteins strongly contribute to microcrystalline cellulose formation in *Komagataeibacter*.

The abundance of BcsAB2 as well as BcsC2 was clearly lower than the homologous proteins expressed from the major operon in *K.* *hansenii* ATCC 53,582 (Fig. 5). Despite being a minor component of the proteome, the significant importance of the minor CS BscAB2 for cellulose fibril synthesis was demonstrated in this study. It was not possible to identify any cellulose modifications such as acetyl groups by FTIR analysis that were possibly introduced by the predicted acetyl transferase (BcsY) encoded within the BcsAB2-containing minor CS operon. Although BcsY was only detected in the proteome of the Δ*bcsAB2* Δ*bcsAB3* double mutant, deletion of the *bcsY* gene led to an altered fiber formation as observed by SEM (Fig. 3) as well as a significant increase of the Young’s modulus (Table 3). BcsX represented a large portion of the proteins expressed from the BcsAB2-containing CS operon, but the role of this uncharacterized SGNH/GDSL hydrolase family protein for BC formation is not known yet. The fact that *bcsAB3* transcription was detected (merely ~ 15% of the BcsAB1 transcript level), but that the corresponding BcsAB3 protein was not detected in our proteome analysis may be caused by its abundance below the detection limit. Nevertheless, a small amount of the protein must be present, since alterations of the BC phenotype were observed by SEM analysis when *bcsAB3* was deleted (Fig. 3). The proteins phosphoglucomutase and UDP-glucose pyrophosphorylase which are involved in forming UDP-glucose, the precursor used for cellulose polymerization, remained unchanged in all deletion mutants.

### Physicochemical properties of the cellulose from wild-type and deletion mutants

Physicochemical characterization confirmed the high crystallinity of BC produced by the *K.* *hansenii* ATCC 53,582 wild type (Table 3), even though the measured crystallinity index (CI) was slightly lower than previously reported (Fang and Catchmark 2015; Wang et al. 2018). We attribute these differences to variations during cultivation or the different evaluation methods. Deletion of the CS gene *bcsAB3* and the simultaneous deletion of *bcsAB2* and *bcsAB3* as well as the deletion of *bcsY* revealed a slight, but significant decrease of the CI, while the single deletion of *bcsAB2* induced only a slight reduction of the measured CI value.

The Young’s modulus is an important rheological parameter to characterize the native tensile strength of the cellulose sheets and to test how the gene deletions of this study affected this property of the cellulose. Deletions generated in the minor *bcsAB2*-containing CS operon (deletions of *bcsAB2* or *bcsY*) had a strong impact, increasing the tensile strength of BC significantly (Table 3). A comparable increase was observed after simultaneous deletion of *bcsAB2* and *bcsAB3*. Since single deletion of *bcsAB3* resulted in a slight decrease in the tensile strength, we assume that the strong increase of the measured Young’s Modulus observed in the Δ*bcsAB2* Δ*bcsAB3* double mutant can be attributed mainly to the deletion of *bcsAB2*. These results support the hypothesis that BcsAB2 and its accessory proteins (BcsX and BcsY), located in the same CS operon, have a strong modifying effect on the assembly of the microfibrils synthesized by BcsAB1. As a working hypothesis, we assume that the CS operons containing BcsAB2 and BcsAB3 may synthesize a different type of cellulose intercalating between the fibers during macrofibril formation from fibers produced by BcsAB1. Alternatively, these cellulose species may be “gluing” together several microfibrils by coating the existing fibers resulting in larger fibrils. We also observed an increase of the mechanical strength after deletion of *dgcA* (Table 3), although the dry mass of the cellulose formed decreased substantially compared to the wild type and the CS deletion strains. The altered mechanical properties resulting from genetic modifications could be an interesting alternative to chemical modification of BC associated with negative environmental effects (Knöller et al. 2020; Sommer et al. 2021). BC with high tensile strength could be of particular interest for applications in tissue engineering and tackle manifold challenges like diffusion, robustness, and biocompatibility (Griffith and Naughton 2002).

Although the BCs of all strains were characterized by a high water content (> 99%), slight differences in water absorption (WAR) were observed (Table 3). The simultaneous deletion of *bcsAB2* and *bcsAB3* resulted in a significantly lower water content compared to the wild type. This effect seems to be in accordance with the impressions from the SEM micrographs that showed an altered fiber formation, when only BcsAB1 is expressed (Fig. 3). The effect of modifications that resulted in an altered water holding capacity could be interesting for food technology to improve its use as thickener, gelling agent, or stabilizer (Cazón and Vázquez 2021; Lin et al. 2020). Until now, only few studies focused on engineering the water-related parameters of BC (Drozd et al. 2021; Fijałkowski et al. 2017).

The results from our study illustrate possible targets for engineering the properties of BC by genetic modification of BC producing strains. It may be rewarding to tailor the mechanical properties by genetic modifications to create BC with novel biotechnologically relevant characteristics.

### Motility assay

BC and biofilm formation in general are linked to the intracellular second messenger cyclic di-GMP. To gain a better understanding of the regulatory mechanism of cellulose synthesis, deletions of the DGCs *dgcA* and *dgcB* were constructed. An enhanced knowledge of the regulation could be used to further increase the cellulose yield. The analysis of the contribution of the DGCs on cellulose formation and on cell motility suggests a more complex regulatory mechanism in *K.* *hansenii* ATCC 53,582 than assumed before. Our results propose an effect on cellulose formation and cell motility different to what was reported previously (Jacek et al. 2019; Tal et al. 1998). Generally, a low cyclic-di-GMP concentration corresponds to reduced biofilm formation, but increased cell motility and vice versa (Schirmer 2016). The deletion of *dgcA* in ATCC 53,582 reduced cellulose formation significantly, while the deletion of *dgcB* totally abolished cellulose synthesis (Fig. 2). On the other hand, the depleted cellulose formation after deletion of *dgcB* did not result in an increased swimming activity of the cells. Instead, the motility of the mutant was significantly reduced compared to the wild type that can spread widely on soft agar under the same conditions. Also, the lack of *dgcA* generated a phenotype incapable of surface swimming. This data is in favor of a local regulatory model of cyclic-di-GMP signaling (Dahlstrom and O'Toole 2017), i.e., cyclic-di-GMP synthesized by DgcB may locally regulate *bcsAB1* by directly binding the PiLZ-domain of *bcsA,* but not via a shared cellular pool of this second messenger. A local regulatory mechanism of cellulose and biofilm synthesis was recently described in detail for *E. coli* (Jenal 2013; Lindenberg et al. 2013; Pfiffer et al. 2019) and may serve as a model for the complex regulation of cellulose synthesis in *K.* *hansenii* ATCC 53,582. A more complete picture of the regulation of cellulose formation may provide the foundation to construct strains with increased cellulose formation.

Although initial genetic studies of BC synthesis were based on transposon mutants, the markerless deletion system for a high-yield cellulose-producing acetic acid bacterium has advantages over transposon mutagenesis as it avoids possible polar effects and allows better biotechnological tuning of BC production in the future. Our work identified the dominant role of BcsAB1 for formation of microcrystalline fibers in the high cellulose-producing strain *K.* *hansenii* ATTCC 53,582. Moreover, the modification of the diameter of cellulose fibers and their mechanical characteristics by BcsAB2 and to a smaller degree by BcsAB3 were described. No amorphous EPS was observed, unlike the nfEPS reported for the closely related strain *K.* *hansenii* ATCC 23,769. However, the SEM micrographs and the tensile strength measurements provide clear indications for the modification of the BC fibers by the minor CS operon containing BcsAB2. The third operon containing BcsAB3 seems to be of little importance for the amount of cellulose synthesized, but shows effects on fiber formation detectable by SEM. The high protein expression of the accessory proteins expressed together with the major operon containing BcsAB1, indicates their importance for the formation of microcrystalline cellulose. The respective cellulose synthase operons will be targets for future studies and strategies to tailor BC properties to suit biotechnological, biomedical, and food technology requirements.

## Supplementary Information

Below is the link to the electronic supplementary material.

Supplementary file1 (PDF 1264 KB)

## Data Availability

The mass spectrometry proteomics data have been deposited to the ProteomeXchange Consortium via the PRIDE partner repository with the dataset identifier PXD037699.
