# Supplementary material for: Analysis of cellulose synthesis in a high-producing acetic acid bacterium Komagataeibacter hansenii
Source: Appl Microbiol Biotechnol. 2023 Mar 17;107(9):2947–67. doi: 10.1007/s00253-023-12461-z (PMC10106347; doi:10.1007/s00253-023-12461-z)
Supplement: Supplementary file 1 — Supplementary file1 (PDF 1264 KB) [file 253_2023_12461_MOESM1_ESM.pdf]

Applied Microbiology and Biotechnology

Genetic and Proteomic Analysis of Cellulose Synthesis in the High-Producing Acetic Acid Bacterium *Komagataeibacter hansenii* ATCC 53582

Martin Bimmer[1], Martin Reimer[2], Andreas Klingl[3], Christina Ludwig[4], Cordt Zollfrank[2], Wolfgang Liebl[1], Armin Ehrenreich[1*]

[1]Chair of Microbiology, Technical University of Munich, School of Life Sciences, Emil-Ramann-Straße 4, 85354 Freising, Germany

[2]Chair for Biogenic Polymers, Technical University of Munich, Campus Straubing, Schulgasse 16, 94315 Straubing

[3]Plant Development, Biozentrum, Ludwig-Maximilans-Universität München, Großhaderner Str.2, 82152 Planegg-Martinsried

[4]Bavarian Center for Biomolecular Mass Spectrometry (BayBioMS), Technical University of Munich, School of Life Sciences, Gregor-Mendel-Straße 4, 85354 Freising, Germany

[*]Corresponding author, aehrenr@tum.de, Phone: +49 (8161) 71 – 5453, Fax: +49 (8161) 71 – 5475

<u>Supplementary figures</u>

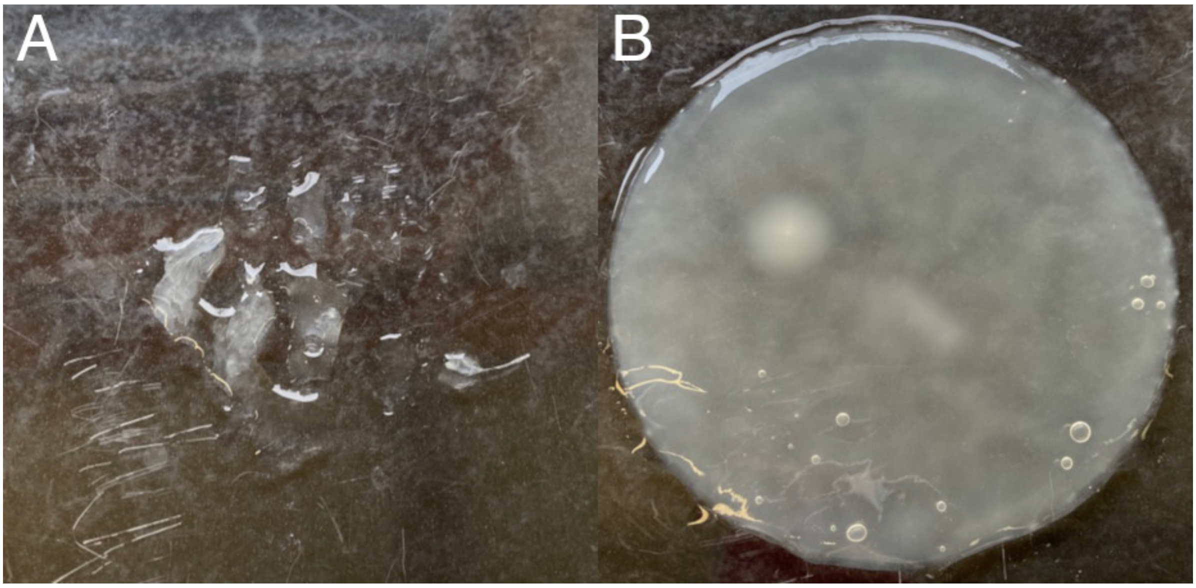

Figure S1. Comparison of cellulose synthesis of *K. hansenii* ATCC 23769 (A) and the high-producing strain *K. hansenii* ATCC 53582 (B) after 6 days of static cultivation on HS medium. Dry weight was measured as described for all *K. hansenii* ATCC 53582. (A) not determinable; (B) 1.62 g/L. The cellulose mass increased after an extended incubation time of 21 days. For *K. hansenii* ATCC 23769 740 mg/L were measured and for *K. hansenii* ATCC 53582 1,8 g/L.

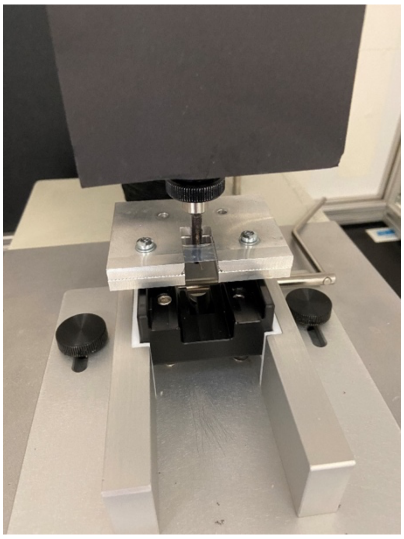

Figure S2. Apparatus for measurement of tensile strength of bacterial cellulose. Self-constructed mount optimized for a Kieffer Rig.

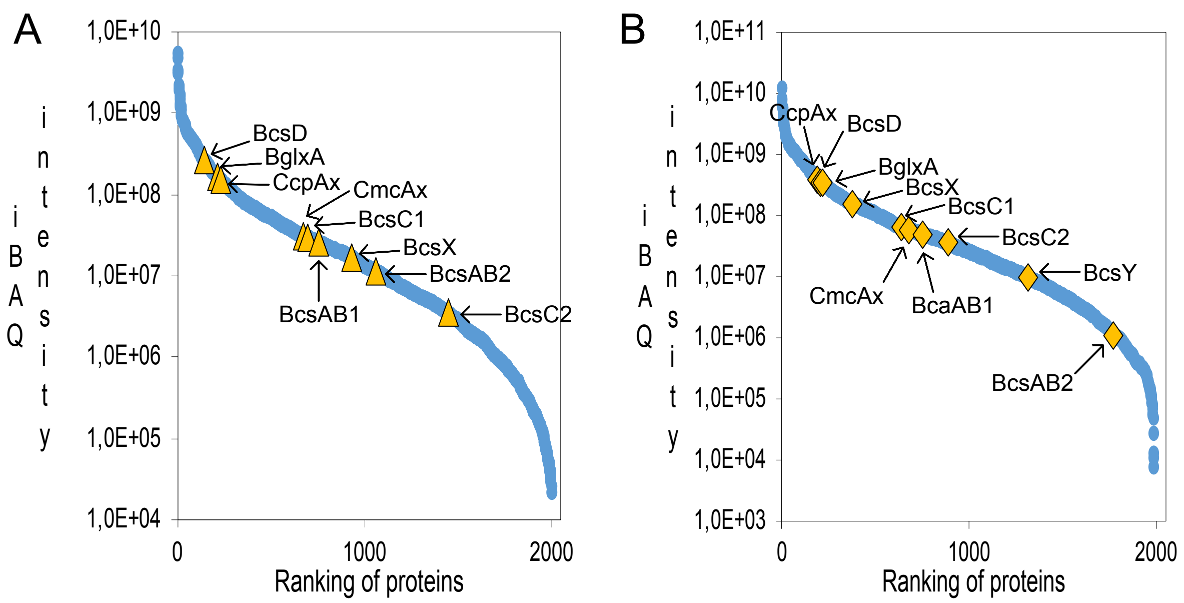

Figure S3. Distribution of the protein concentrations measured for all detectable proteins in K. hansenii ATCC 53582 (A) and the double deletion strain K. hansenii ΔbcsAB2 ΔbcsAB3 (B) using LC-MS/MS-based proteomics. Order of the proteins in wildtype (yellow triangles from left to right): BcsD, BglxA, CcpAx, CmcAx, BcsC1, BcsAB1, BcsX, BcsAB2, BcsC2. Order of the detected proteins in double deletion mutant (yellow rhomb from left to right): CcpAx, BcsD, BglxA, BcsX, BcsC1, CmcAx, BcsAB1, BcsC2, BcsY, BcsAB2.
